# The First Call Note Plays a Crucial Role in Frog Vocal Communication

**DOI:** 10.1038/s41598-017-09870-2

**Published:** 2017-08-31

**Authors:** Xizi Yue, Yanzhu Fan, Fei Xue, Steven E. Brauth, Yezhong Tang, Guangzhan Fang

**Affiliations:** 1 Department of Herpetology, Chengdu Institute of Biology, Chinese Academy of Sciences, No.9 Section 4, Renmin Nan Road, Chengdu, Sichuan P. R. China; 2 University of the Chinese Academy of Sciences, 19A Yuquan Road, Beijing, P. R. China; 3 Department of Psychology, University of Maryland, College Park, MD 20742 USA

## Abstract

Vocal Communication plays a crucial role in survival and reproductive success in most amphibian species. Although amphibian communication sounds are often complex consisting of many temporal features, we know little about the biological significance of each temporal component. The present study examined the biological significance of notes of the male advertisement calls of the Emei music frog (*Babina daunchina*) using the optimized electroencephalogram (EEG) paradigm of mismatch negativity (MMN). Music frog calls generally contain four to six notes separated approximately by 150 millisecond intervals. A standard stimulus (white noise) and five deviant stimuli (five notes from one advertisement call) were played back to each subject while simultaneously recording multi-channel EEG signals. The results showed that the MMN amplitude for the first call note was significantly larger than for that of the others. Moreover, the MMN amplitudes evoked from the left forebrain and midbrain were typically larger than those from the right counterpart. These results are consistent with the ideas that the first call note conveys more information than the others for auditory recognition and that there is left-hemisphere dominance for processing information derived from conspecific calls in frogs.

## Introduction

Acoustic signaling is critically important for both survival and reproductive success in vocalizing animals. Vocalization usually provides diverse information including the signaler’s species, individual identity, reproductive status, location and resources occupation^1^. Communication sounds may also reflect differences in the behavioral states of individuals including reproductive state, territorial defense, foraging and anti-predation^2, 3^. Animal vocalizations thus serve as complex signals encoding multiple forms of information^4–6^. Nevertheless, the manner in which each kind of information is encoded in complex signals is largely unknown. Furthermore the time-frequency properties of animal vocalizations^7–11^ as well as the structure of vocalizations (notes, elements or syllables)^12–14^ usually differ significantly across individuals consistent with the idea that these signals are important cues for species discrimination and individual recognition. Taken together these findings imply that each component of such complex animal vocalizations may have distinct functions and biological significance.

Most anuran species relay almost completely on sound communication for reproductive success. Anuran calls usually consist of a series of notes and intervals. For example, male Emei music frogs (*Babina daunchina*) call from underground burrows that serve as nests for egg laying and tadpole development or in open fields. Behavioral studies show that male calls produced from inside burrows are modified acoustically by the resonant properties of the burrows and are both more highly sexually attractive to females and evoke stronger competitive responses from rival males^15, 16^. Male calls thus provide information related to both male-male competition and female choice. Interestingly, female low amplitude click-like calls can serve to incite males to increase competitive behaviors^17^. In music frogs most advertisement calls are composed of four to six notes with around 150 ms intervals and these notes usually exhibit significantly different time-frequency properties^18^. The present study was designed to use the Emei music frog as a model for investigating how call components, in this case the call notes, may differ functionally.

The event-related potential (ERP) is a measured electrophysiological brain response consisting typically of a series of positive and negative waves which is time-locked with stimulation by a specific sensory, cognitive or motor event^19^. In humans, auditory mismatch negativity (MMN) is a change in the amplitude and/or latency of a late negative ERP component with a frontal-central distribution, which occurs in response to an unexpected (deviant) auditory stimulus inserted in a series of standard stimuli^19^. It is believed to reflect increased processing.

The biological mechanism underlying MMN is believed to be that of an automatic switch directing the listener’s attention to the unpredictable acoustic novel deviant stimulus involving sensory-memory updating and alarming functions^11, 20^. MMN amplitude may therefore indicate the resources and energy devoted to the related cognitive processing. Previous research in *Babina* has shown that male calls produced from inside burrows (which are highly attractive to females and engender high competitive responses from males) yield significantly different electroencephalogram (EEG) or ERP patterns compared to those produced outside burrows^21–23^. Previous ERP research has also shown that call differences reflecting sound recognition and call discrimination are decoded in order^22^. Thus it is reasonable to ask if auditory processing differs between successive call notes as would be reflected by different ERP MMN amplitudes. In addition to these considerations, behavioral and EEG studies also indicate that music frogs manifest right-ear/left-mesencephalon advantage in processing species-specific vocalizations^21, 24, 25^, suggesting that MMN amplitude distributions would also be lateralized.

In view of the above described results of previous studies we predicted that 1) different advertisement call notes with differing dynamic time-frequency properties would induce significantly different MMN amplitudes and that the first note would evoke the largest amplitude; and 2) a left brain biased lateralization would exist for the MMN distributions. To test our predictions, we recorded multi-channel electrocorticogram (ECoG) signals from *B. daunchina* in response to different call notes in order to assess the biological significance and salience of the time-frequency properties of each note.

## Results

**Figure 1:**
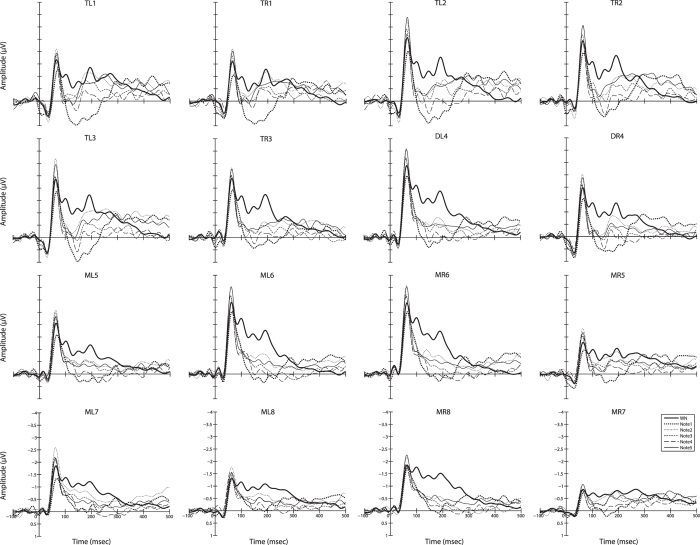
The MMN amplitudes of the original waves for the standard stimulus and the five call note deviant stimuli with respect to each channel. Abbreviation: WN and Note1/2/3/4/5 denote the original waves for the white noise and the five notes respectively; TL1-3 and TR1-3, the six channels on the telencephalon; DL4 and DR4, the two channels on the diencephalon; ML5-8 and MR5-8, the eight channels on the mesencephalon.

**Figure 2:**
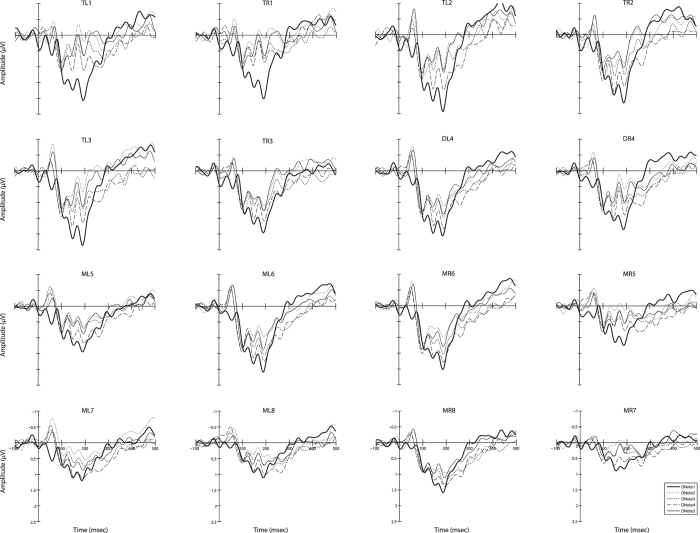
The amplitudes of the difference waves for the five call note deviant stimuli with respect to each brain area. Abbreviation: DNote1/2/3/4/5 denote the difference waves for the five notes respectively; TL1-3 and TR1-3, the six channels on the telencephalon; DL4 and DR4, the two channels on the diencephalon; ML5-8 and MR5-8, the eight channels on the mesencephalon.

The grand average of the original waves and difference waves for each acoustic stimulus and each EEG channel are presented in Figs 1 and 2 respectively. Visual inspection for all difference waves reveals a positive-going deflection occurring between 50 and 250 ms, which is defined as the MMN component in humans^26^ and that has been taken to indicate auditory system processing for automatically detecting vocal deviants. Statistical analysis reveals that significant differences in amplitudes but not latencies exist between stimuli and EEG channels respectively as elucidated below. With respect to the MMN latencies for different brain regions (telencephalon, diencephalon and mesencephalon), there was no significant main effect or interaction for any factors.

### MMN amplitudes for the telencephalon

**Figure 3:**
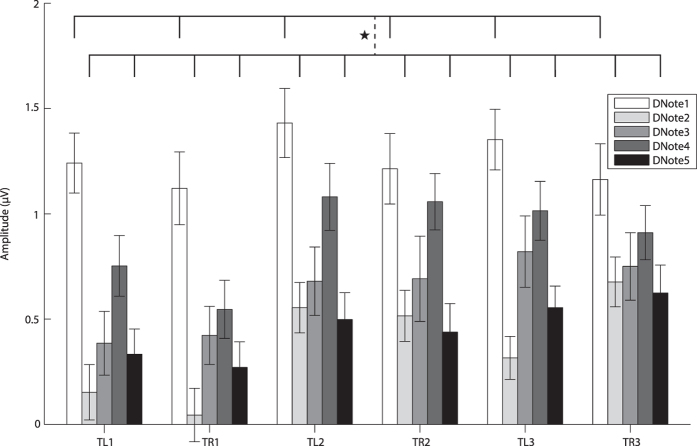
The average amplitudes and standard errors of MMN difference waves for the telencephalon. The vertical dashed line denotes a significant difference between the two groups connected by the line. Abbreviation: DNote1/2/3/4/5 denote the MMN amplitudes of the difference waves for the five notes, respectively; TL1, TR1, TL2, TR2, TL3 and TR3 represent the electrodes on the telencephalon.

Analysis of telencephalic MMN amplitudes shows that the main effects for the factors “acoustic stimulus” (F (4, 44) = 2.818, partial *η* ^2^ = 0.204, *p* = 0.036) and “EEG channel” (F (5, 55) = 2.692, partial *η* ^2^ = 0.197, *p* = 0.030) were significant. The trend of MMN amplitudes elicited by the five notes shows that DNote1 > DNote4 > DNote3 > DNote5 > DNote2. Multiple comparisons showed that the MMN amplitude elicited by Note1 was significantly larger than those by Note2 and Note5 (*p* < 0.05, Fig. 3), implying Note1 might play a key role in vocal communication. The MMN amplitudes for the six electrodes exhibited the following trend across all notes: TR3 > TL2 > TR2 > TL3 > TL1 > TR1. For MMN amplitudes, TL2 was significantly larger than TL1 and TR1, while TL3 was significantly larger than TR1 (*p* < 0.05, Fig. 3). Overall, the MMN amplitudes for the left channels were larger than those for the right counterparts except for the caudal pair of electrodes (TL3 and TR3), consistent with the idea that right-ear/left-hemispheric advantage exists for processing communication sounds in frogs. As for the MMN amplitudes on the same side of telencephalon, the posterior electrode values were always larger than those of the anterior ones: TR2 and TR3 were significantly larger than TR1 (*p* < 0.05, Fig. 3).

### MMN amplitudes for the diencephalon

**Figure 4:**
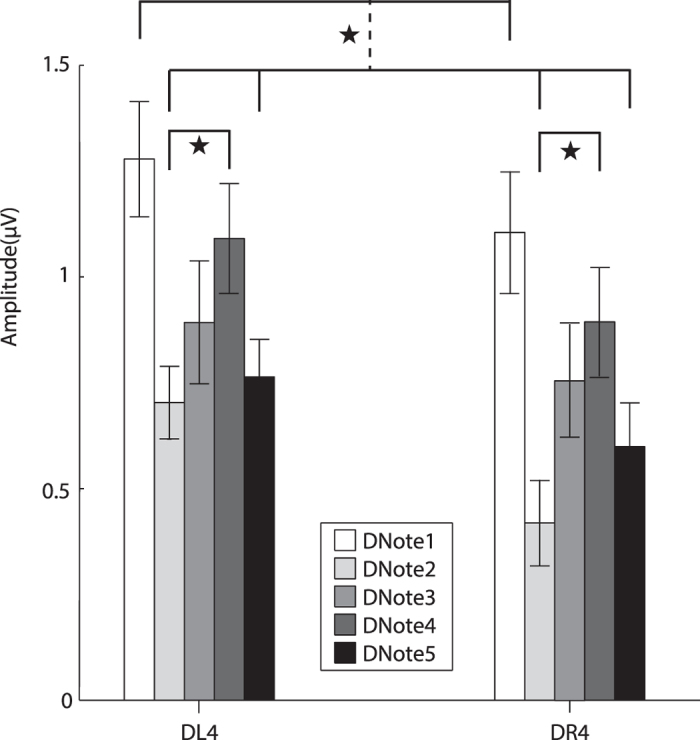
The average amplitudes and standard errors of MMN difference waves for the diencephalon. The vertical dashed line denotes a significant difference between the two groups connected by the line. Abbreviation: DL4 and DR4 represent the electrodes on the diencephalon.

The analysis of the MMN amplitudes in the diencephalon showed a significant main effect for the factor “acoustic stimulus” (F (4, 44) = 2.531, partial *η* ^2^ = 0.187, *p* = 0.054) but not the factor “EEG channel” (F (1, 11) = 1.884, partial *η* ^2^ = 0.146, *p* = 0.197). The MMN amplitudes elicited by the five notes exhibited the same trend as the telencephalon, i.e. DNote1 > DNote4 > DNote3 > DNote5 > DNote2. Multiple comparisons showed that the MMN amplitude elicited by Note1 was significantly larger than those of Note2 and Note5, while the MMN amplitude for Note4 was significantly larger than that of Note2 (*p* < 0.05, Fig. 4). Thus, these results imply that for the diencephalon Note1 plays a different role in vocal communication compared with the other call notes. Moreover, the MMN amplitudes for the left channel were larger than those of the right counterpart although the differences did not reach statistical significance.

### MMN amplitudes for the mesencephalon

**Figure 5:**
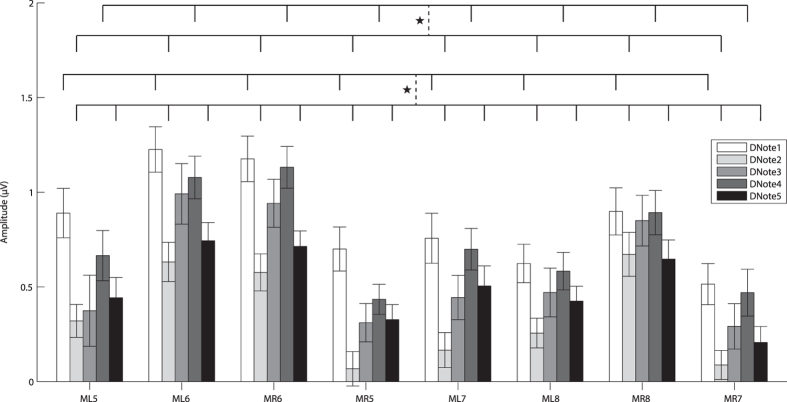
The average amplitudes and standard errors of MMN difference waves for the mesencephalon for each call note. Each vertical dashed line denotes a significant difference between the two groups connected by the line. Abbreviation: ML5, MR5, ML6, MR6, ML7, MR7, ML8 and MR8 represent the electrodes on the mesencephalon.

The analysis of the MMN amplitudes for the mesencephalon showed significant main effects for the factors “acoustic stimulus” (F (4, 44) = 2.654, partial *η* ^2^ = 0.194, *p* = 0.045) and “EEG channel” (F (7, 77) = 3.720, ε = 0.490, partial *η* ^2^ = 0.253, *p* = 0.016). The MMN amplitudes elicited by the five notes exhibited trend similar to those of both the telencephalon and diencephalon. The MMN amplitude elicited by Note1 was significantly larger than those elicited by Note2 and Note5, while the MMN amplitude for Note4 was significantly larger than that of Note2 (*p* < 0.05, Fig. 5). The MMN amplitudes for the eight electrodes exhibited the trend pattern ML6 > MR6 > MR8 > ML8 > ML5 > ML7 > MR7 > MR5. Multiple comparisons showed that the MMN amplitudes for ML6 and MR6 were significantly larger than those of ML5, MR5, ML7, MR7 and ML8 (*p* < 0.05, Fig. 5). As for the forebrain electrodes, the results for the midbrain showed that the MMN amplitudes for the left channels were larger than those for the right counterparts except for the caudal pair of electrodes (ML8 and MR8). The MMN amplitudes of the electrodes located on the central mesencephalon were always larger than those on the lateral mesencephalon: the MMN amplitude for ML8 and MR8 were significantly larger than those for MR5 and ML7 (*p* < 0.05, Fig. 5).

### Results for N1, P2 and P3

The grand averages of the original waveforms and difference waveforms are presented in Figure S1 in the Appendix. For N1, the amplitudes of the OT (the original envelope for which only the temporal properties are preserved) were significantly higher than those of the ON (original note) and OS (reversed version of the ON for which the spectral properties remained unchanged) regardless of brain regions (*p* < 0.05, Table 1 and Figure S2 in the Appendix). For P2, the ON and OS amplitudes were significantly higher than those of the OT regardless of brain regions, although the difference between the ON and OT did not reach statistical significance for the telencephalon (*p* < 0.05, Table 1 and Figure S3 in the Appendix). Moreover, the amplitude for TR2 was significantly higher than that for TR1, while the amplitudes for TR2 and TR3 were significantly higher than those for TL1 and TL3. For P3, the amplitudes for the ON and OS were higher than that for the OT regardless of brain regions, although the difference between the ON and OT did not reach statistical significance for each brain region (*p* < 0.05, Table 1 and Figure S4 in the Appendix). There was no significant difference for the factor “EEG channel”.

**Table 1:** Results of ANOVAs for the amplitudes of N1, P2 and P3 with respect to two factors for the telencephalon, diencephalon and mesencephalon respectively.

	for the telencephalon/(2,22), (5,55), (10,110)					for the diencephalon/(2, 22),(1, 11),(2, 22)					for the mesencephalon/(2, 22),(7, 77),(14, 154)				
	*F*	**ε**	*p*	*η* ^2^	LSD	*F*	**ε**	*p*	*η* ^2^	LSD	*F*	**ε**	*p*	*η* ^2^	LSD
*N1*															
stimulus	5.866	0.658	0.022^*^	0.348	OT > ON,OS	12.665	NA	0.000^**^	0.535	OT>ON,OS	9.599	NA	0.001^*^	0.466	OT>ON,OS
channel	0.466	0.490	0.671	0.041	NA	0.031	NA	0.864	0.003	NA	0.427	0.327	0.684	0.037	NA
interact	0.585	0.343	0.650	0.051	NA	0.614	NA	0.550	0.053	NA	1.388	NA	0.165	0.112	NA
*P2*															
stimulus	3.438	NA	0.050^#^	0.238	OS>OT	6.080	0.673	0.019^*^	0.356	ON,OS>OT	4.622	NA	0.021^*^	0.296	ON,OS>OT
channel	3.302	0.426	0.052^#^	0.231	TR2>TR1	0.035	NA	0.855	0.003	NA	0.694	0.366	0.542	0.059	NA
				TR2,TR3>TL1,TL3											
interact	1.217	0.274	0.319	0.100	NA	1.649	NA	0.215	0.130	NA	0.828	NA	0.638	0.070	NA
*P3*															
stimulus	5.703	0.685	0.031^*^	0.316	OS>ON	6.432	0.683	0.016^*^	0.369	OS>ON,OT	5.423	NA	0.012^*^	0.330	OS>ON,OT
channel	2.341	0.456	0.111	0.175	NA	0.258	NA	0.622	0.023	NA	1.479	0.416	0.239	0.119	NA
interact	1.175	0.242	0.331	0.096	NA	1.493	0.601	0.249	0.120	NA	0.597	NA	0.864	0.051	NA

Note: The symbols ‘>’ denote that the amplitudes of the given ERP component and given brain region for the acoustic stimulus or EEG channel on the left side of ‘>’ are significantly larger than those on the right side, and no significant difference exists among the corresponding conditions on the same side of ‘>’ for each case. The degrees of freedom are shown after the brain regions for the two factors and their interaction respectively. ^#^0.05 < *p* < 0.1, *p < 0.05, **p < 0.001. Abbreviations: *F* is the *F*-value from ANOVA; ε, the values of epsilon of Greenhouse-Geisser correction; LSD, least-significant difference test; ON, original note; OS, reversed version of the original note (only spectral characteristics remained); OT, white noise enveloped by the original note (only temporal characteristics remained); TL1–3 and TR 1–3, the six channels on the telencephalon.

## Discussion

The results of the present study show that when the six acoustic stimuli, consisting of WN and the five notes, are presented according to the optimum paradigm of MMN, 1) the MMN amplitudes evoked by the first note are greater than that of the other notes in the telencephalon, diencephalon and mesencephalon; and 2) the MMN amplitudes evoked from the left channels are greater than those of the right channels. These results are consistent with the hypothesis that the first note plays unique and presumably critical role in anuran vocal communication, and that the brain exhibits left biased lateralization for processing these acoustic stimuli. Furthermore, comparison of the responses evoked by the note envelope with those evoked by the original note retaining all temporal properties, and the note stimuli with reversed temporal properties reveals that ERP components are most similar between the original note and reversed note. These results support the idea that the spectral characteristics of the stimuli are of key importance in vocal communication.

Different components of acoustic signals may function differently for vocal communication. In zebra finch ‘micro-structural’ variation (i.e. syllable type) influences mate choice within subspecies and ‘macro-structural’ variation (i.e. spectral and temporal characteristics) determine mate choice between subspecies^27, 28^. In *Gekko gecko*, phase I and phase II of advertisement calls are reported to function for individual identification and species recognition, respectively^14^. The present study reveals that significant differences in MMN amplitudes occur in response to Note1 stimulation compared to the other notes in music frogs. Since MMN amplitudes always reflect differences between a standard and deviant stimuli^29^, MMN amplitudes have been taken to reflect brain resources devoted to cognitive processes^26, 29, 30^. For this reason it is reasonable to speculate that the first note in music frog calls is the most important unit for vocal communication. This idea is supported by a previous study which showed that the first note plays a critical role in anurans’ sound recognition and call discrimination^22^.

A temporal hierarchy for neural processing of information contained in advertisement calls has been described in the music frog insofar as sound classification and call discrimination are accomplished in ~100 ms and in ~200 ms, respectively^22^. A similar temporal hierarchy exists in humans who discriminate vocalizations produced by living versus man-made sources in 70–119 ms, human vocalizations from animal vocalizations in 169–219 ms and musical from non-musical sounds in 291–357 ms^12^. In music frogs, the first note generally lasts about 200 ms. Notably, species recognition and individual discrimination are achieved within this period insofar as discrimination of calls produced by the same male from inside or outside burrows takes ~200 ms. The precedence effect is an inherent response property of the vertebrate auditory system^31, 32^. Consequently, the first call note must encode the most crucial information for the species recognition, individual discrimination and call type identification.

For males of most anuran species, calling activity is also energetically costly and may attract potential predators in addition to potential mates. Consequently, encoding the most important information in the initial call period would not only facilitate species discrimination and individual recognition by females but would also reduce the signaler’s predation risks. The present results also show that the second call note elicits significantly smaller MMNs than the other notes, consistent with the idea that the second note is a comparatively less important functional call component.

Masking noises can interfere with the perception of acoustic signals by receivers and distract attention^33, 34^. Although nothing is known about forward or backward masking in frogs^35^, females in *Kassina fusca* prefer the following call of one male or the leading call from another based on the time elapsed between the calls^36^. Thus it seems reasonable to assume that backward masking on the leading call can be produced by the first note of the following call in anuran receivers if the inter-note interval is within a sufficiently short time window. Accordingly, males may be able to achieve advantages over rivals in male-male competition and female attraction, by minimizing the masking effects of the second note on the first note. The smaller MMN amplitude and presumed reduced importance of Note2 stimulation might reflect reduced masking potential of this note.

The analysis of call acoustic characters using multidimensional scaling shows that the temporal and spectral properties of the first note are distinctively different from those of the other call notes^18^. It is logical to infer that MMN amplitude is highest for the first call note because these acoustic features exist. Studies of the acoustic structure and time-frequency properties of bird vocalizations have also been shown to be important for species recognition and individual discrimination^27, 28, 37^. In these species MMN amplitudes elicited by auditory stimuli may be highly dynamic with peak amplitude increasing as stimulus frequency increases^38^. In music frogs both the fundamental and dominant frequencies of advertisement calls typically increase from the first to the last note, a pattern human listeners in the Emei Mountain area historically found ‘melodic’^18^. Interestingly, the Note1 exemplar with the lowest frequency profiles evoked the largest MMN amplitude in the present study. Furthermore, the time between the first and second notes typically exceeds 250 ms, the MMN time window. Thus it is reasonable to propose that the first note is poised to provide most of the crucial cognitive cues necessary for the recognition process.

In humans, syllable duration^39^ and number of harmonic bands^40^ are positively correlated with MMN amplitudes. For music frogs the durations of the five call notes were 170, 108, 93, 94 and 95 ms and the number of harmonic bands were 7, 5, 5, 5 and 4, respectively. Nevertheless the fourth note, with short duration and few harmonics evoked the second largest MMN amplitude. Clearly in music frogs call unit durations and number of harmonics are not crucial factors. In humans MMN amplitude is also related to the envelopes of auditory stimuli^41^ as well as frequency complexity^42^. In music frogs the largest MMN amplitude of Note1 might thus reflect the combined effect of the particular temporal and spectral characteristics including fundamental and dominant frequencies, duration, harmonic and envelope. Consistent with this idea, the present results show that ERP amplitudes evoked by the OT (the envelope of the note retaining only the temporal properties) were quite different from those evoked by the ON (original note) and the OS (the reversed version of the ON with only the spectral properties retained) regardless brain regions, while the amplitudes evoked by the ON and OS were similar. This suggests that although both the temporal and spectral note characteristics are important in vocal communication, the fundamental frequency and correlated note spectral properties are the most individually distinctive call properties in some anuran species^43, 44^.

In a previous study we found right-ear/left-mesencephalon predominance in anuran acoustic signal processing^21^. The present results show that most MMN amplitudes in response to the five note stimuli were larger in left brain areas than in right brain areas. Given that MMN amplitude reflects the brain resources devoted to signal processing, these results provide more evidence for the idea that the left forebrain and midbrain (including the telencephalon, diencephalon and mesencephalon) are dominant in frogs for the processing of communication sounds. These results are also consistent with many previous studies in land vertebrates indicating a right-ear/left-hemispheric advantage for language in humans^45^ and conspecific communication sounds in nonhuman primates^46, 47^, other mammals^48–50^, birds^51^ and frogs^16, 21, 22, 25, 52^.

In amphibians the midbrain torus semicircularis integrates auditory input from the brainstem and projects to the diencephalon, from which auditory information is projected to the telencephalon^53^. MMN amplitudes recorded from midbrain sites (ML6, MR6, ML8 and MR8) near the midline were larger than those placed laterally, consistent with the idea that these electrodes were placed closer to the torus semicircularis.

For humans and animals, selective attention is essential for individual recognition in noisy environments as evidenced by the ‘cocktail party effect’^54–56^. MMN reflects the automatic detection of stimulus differences and participates in the formation of a standard stimulus template^9, 57, 58^ regardless of the subject’s direction of attention^26, 29, 30^. Nevertheless the auditory context is the primary modulator of the MMN and can be influenced by both stimulus-driven and attentional factors^59^. The present study sheds light on how stimulus-driven factors affect the MMN in frogs although, as yet, the role of attention remains an open question for further investigation.

## Materials and Methods

### Animals and surgery

Twelve (12) adult frogs (6 males and 6 females) obtained from the Emei mountain area of Sichuan, China were used in the experiments. Animal husbandry, laboratory animal care and EEG experimental recording procedures were the same as used in previous work and have been described elsewhere^22^. Briefly, the subjects were segregated by sex in separate opaque plastic tanks (45 × 35 cm^2^ and 30 cm deep) which contained mud and water, and maintained on a 12:12 light-dark cycle (lights on at 08:00). The tanks were placed in a room under controlled temperature conditions (23 ± 1 °C) and fed fresh live crickets every three days. Mean mass was 11.1 ± 2.4 g and length was 4.6 ± 0.3 cm at the time of surgery. Permits were obtained from Department of Forestry Management, Mount Emei and Leshan Giant Buddha Management Committee. The experimental protocols were approved by the Animal Care and Use Committee of Chengdu Institute of Biology under the license number 2016005 and carried out according to the international standards of animal care and use. All surgery was performed under metacaine (MS-222) anesthesia, and all efforts were made to minimize discomfort.

**Figure 6:**
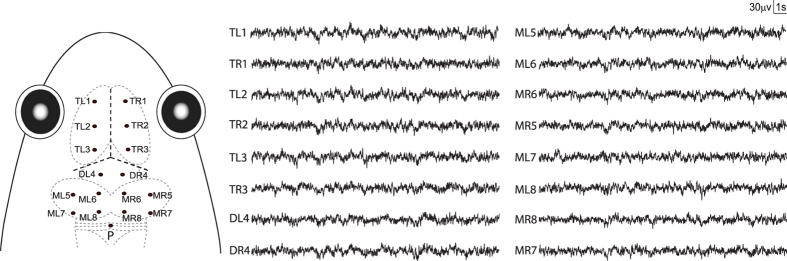
Electrode placements and 20 sec of typical EEG tracings obtained from each. The intersection of the three dashed lines in bold in the frog head denotes the intersection of suture lines corresponding to lambda. The electrodes coordinates: TL1 (−1.5, 3.8), TR1 (1.5, 3.8), TL2 (−1.5, 2.4), TR2 (1.5, 2.4), TL3 (−1.5, 1), TR3 (1.5, 1); DL4 (−0.8, −0.2), DR4 (0.8, −0.2); ML5 (−2.2, −1.6), ML6 (−0.8, −1.6), MR6 (0.8, −1.6), MR5 (2.2, −1.6), ML7 (−2.2, −3.5), ML8 (−0.8, −3.5), MR8 (0.8, −3.5), MR7 (2.2, −3.5); P (0, −4.5).

All experiments were performed during the reproductive season of this species. Briefly, after anesthetizing the subject using a 0.15% MS-222 solution^60^, seventeen cortical EEG recording electrodes, consisting of miniature stainless steel screws (φ 0.5 mm), were implanted on the skull. Sixteen (16) electrodes were distributed in the left and right sides of telencephalon (TL1, TR1, TL2, TR2, TL3, TR3), diencephalon (DL4, DR4) and mesencephalon (ML5, MR5, ML6, MR6, ML7, MR7, ML8, MR8). The reference electrode (P) was placed on the cerebellum (Fig. 6). All electrode leads were formvar-insulated nichrome wires with one end interwined tightly around the screws and the other end tin soldered to the female-pins of an electrical connector. Electrodes were fixed to the skull with dental acrylic. The connector was covered with a self-sealing membrane (Parafilm^®^ M; Chicago, USA) that was water-proof and located about 1 cm above the head of the animal. Each frog was housed individually for 6 days for recovery before conducting further experiments. After all experiments were completed, the subjects were euthanized by overdose of MS-222 and electrode localizations were confirmed by injecting hematoxylin dye through the skull holes in which the electrodes were installed previously (see Figure S5 in the Appendix).

### Recording conditions

An opaque plastic tank (80 × 60 cm^2^ and 60 cm deep) containing mud and water was placed in a soundproof and electromagnetically shielded chamber (background noise 24.3 ± 0.7 dB). An infrared camera with a motion detector was mounted centrally about one meter above the tank for monitoring the subjects’ behaviors. Electrophysiological signals were recorded with a signal acquisition system (OmniPlex 64-D, Plexon, USA). The sampling rate was 1000 Hz.

### Stimuli

**Figure 7:**
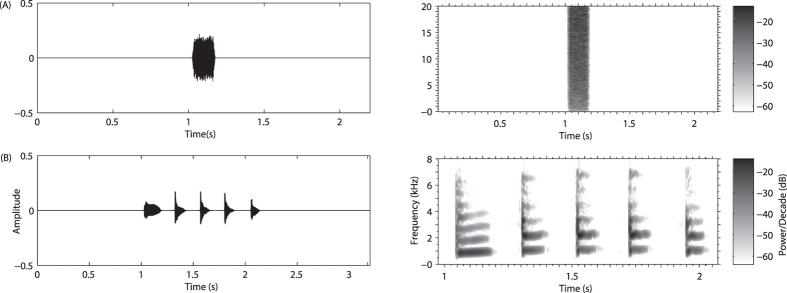
Waveforms and spectrograms of the six stimuli: (**A**) White noise (WN); (**B**) The randomly selected advertisement call notes derived from calls which each contained five notes (Note1, Note2, Note3, Note4 and Note5).

In view of the goal of the experiments and consistent with basic MMN requirements, five successive notes (Note1, Note2, Note3, Note4 and Note5) of a male advertisement call were selected as the deviant stimuli with white noise (WN) used as the standard stimulation. Since the ponds in which the playback signals were recorded were far from the ponds from which the subjects were captured, each subject did not experience the playback stimuli before the experiments. The duration of WN equaled the average duration of the durations of the conspecific call notes with 10 millisecond rise and fall times in sinusoidal periods (Fig. 7). The stimuli were play backed by two speakers (SME-AFS, Saul Mineroff Electronics, Elmont, NY, USA) equidistantly placed at the opposite ends of the experimental tank, and the sound pressure of each stimulus was adjusted to 65 ± 0.5 dB SPL at the center of the tank using a sound pressure meter (relative to 20 μPa, A-weighting; Aihua, AWA6291; Hangzhou, China).

### Preliminary Study to Rule out Pseudoreplication Effects

Because pseudoreplication can affect statistical analysis in ecological, animal behavior and neuroscience studies^61, 62^ we controlled for the possible effects of pseudoreplication on our conclusions in the present study. Four advertisement calls made up of five notes were recorded from four different individuals and used for a preliminary MMN experiment^63^. Each of the five notes derived from each call were played back to four experimental animals. The results showed that there was no significant difference in the MMN responses between the four advertisement calls, and that the first note from each call yielded the MMN of largest amplitude^63^. Since different call notes recorded from different individuals are likely to express information specific to a given individual concerning that individual’s characteristics, pooled data acquired from the calls of four animals would attenuate MMN responses due to the fact that the information contained in the call notes derived from each male would differ. Thus notes from a single call used in the preliminary experiment was selected randomly in the present study in order to determine if note information is associated with the serial position of the note in the call.

### MMN paradigm

MMN responses have usually been evoked by stimulations using the Oddball Paradigm in which subjects detect small numbers of unexpected/novel acoustic stimuli distributed among a larger number of uniform/standard stimuli. Although the insertion of irrelevant stimuli in the stimulation sequence can be used to eliminate the component effect of stimulus expectancy^11, 64^, the Optimum paradigm allows use of an even shorter measurement time without compromising the MMN amplitude, compared with the traditional Oddball paradigm^65^. Thus, the Optimum paradigm was used in the present study. For this paradigm each call note type (Note1/2/3/4/5) served as a deviant stimulus and was randomly inserted after a single standard stimulus (WN) presentation within the stimulation sequence. In other words, all 5 deviants (*p* = 0.1; each) were presented in the same sequence so that every other stimulus was allocated to standards (*p* = 0.5) with one of the 5 deviants inserted between two standard stimuli (see Figure S6 in the Appendix). Theoretically the deviant stimuli would serve to strengthen the memory trace of the standard stimulus in this paradigm and would therefore elicit almost equal MMN waves compared with using the traditional Oddball paradigm^65^.

The stimulation streams contained 1845 stimuli with 500 ms inter-stimulus intervals (ISI). The session lasted about 30 min and was divided into three blocks with 5 min breaks between blocks so that the subjects would not become fatigued^66^. In each block, the first 15 acoustic stimuli were standards; in the following sequence, one of the deviants was presented after each standard stimulus until all five deviant stimuli had been presented once (see Figure S6 in the Appendix). A trigger pulse was sent to the signal acquisition system at every stimulus onset through the parallel port for further time-locking analysis.

### EEG signal collection and MMN measurement

After postoperative recovery for 6 days, the subject was placed in the experimental tank and connected to the signal acquisition system for about 24 hours habituation. Then the EEG signal and behavioral data were collected according to the above described auditory stimulation paradigm. In order to eliminate the effects of digestion, the subject was not fed during the experimental period. Prior to MMN analysis, raw EEG data were filtered by a band-pass filter from 0.25–25 Hz, then partitioned into epochs, inspected and averaged as described in previous studies^26, 65^. The MMN component was defined as the mean amplitude during the interval from 50–250 ms after the onset of stimulation^29, 67, 68^. The amplitude and latency of MMN were calculated by the “average amplitude measure”^69^ and “50 percent area latency measure”^19^ respectively. The mismatch negativity is described as the difference waves calculated by subtracting the ERP amplitudes in response to WN as the standard stimulation from those to conspecific notes as the deviant stimulation^26^. Consequently, the amplitudes and latencies of difference waves rather than those of original waves were further statistically analyzed. The difference waves elicited by Note1/2/3/4/5 were defined as DNote1/2/3/4/5.

### Effects of temporal and spectral characteristics

The traditional Oddball paradigm with a standard and three deviants was used to assess the potential contributions of both the temporal and spectral characteristics of the first call note, and ERP components (N1, P2 and P3) were analyzed. Continuous white noise (WN) with 10 ms rise and fall times in sinusoidal periods was used as the standard in order to eliminate any temporal or spectral characteristics (see Figure S7 in the Appendix). The three deviant stimuli were the advertisement call used for MMN paradigm (original notes, ON), its reversed version with each reversed note (only spectral characteristics remained, OS) and its envelope version filled with white noise (only temporal characteristics remained, OT). The standard and each of the three deviants were presented randomly with a 1.5 s ISI. The probability of appearance for each stimulus was 70% and 10%, respectively. EEG signal collection and ERP component measurement were similar to those for MMN. The time windows for N1, P2 and P3 were chosen as 30–130 ms, 150–250 ms and 250–350 ms, respectively^22^. Only ERP components for the first note were analyzed.

### Statistical analyses

The normality of the distribution and the homogeneity of variances of the MMN values were estimated with the Shapiro-Wilk *W* test and Levene’s test, respectively. Since there were too many EEG channels for statistical analyses, the amplitudes and latencies of MMN and other ERP components were statistically analyzed for the telencephalon, diencephalon and mesencephalon respectively. For each of these brain structures, a three-factor repeated measured ANOVA was conducted with the variables of “sex” (male/female), “acoustic stimulus” (DNote1/2/3/4/5) and “EEG channel” (TL1, TR1, TL2, TR2, TL3 and TR3 for the telencephalon; DL4 and DR4 for the diencephalon; ML5, MR5, ML6, MR6, ML7, MR7, ML8 and MR8 for the mesencephalon). For both amplitudes and latencies of MMN and other ERP components, there were no significant main effects for the factor “sex”. Therefore all datasets were pooled regardless of “sex” and statistically analyzed using a two-way repeated measured ANOVA. Both main effects and interactions were examined. If ANOVAs returned a significant difference, the data was further tested for multiple comparisons using the least significant difference test. If the interaction was significant, simple effects analysis was applied. Greenhouse-Geisser epsilon (ε) values were employed when the Greenhouse-Geisser correction was necessary. Effect size was determined with partial *η* ^2^ (partial *η* ^2^ = 0.20 is set as a small, 0.50 as a medium and 0.80 as a large effect size, respectively)^70^. SPSS software (release 20.0) was applied for the statistical analysis with *p* < 0.05 set as the significance level; *p* values > 0.05 and < 0.1 were considered as marginally significant^71^.

## Electronic supplementary material

Appendix
