# Supplementary material for: The First Call Note Plays a Crucial Role in Frog Vocal Communication
Source: Sci Rep. 2017 Aug 31;7:10128. doi: 10.1038/s41598-017-09870-2 (PMC5579009; doi:10.1038/s41598-017-09870-2)
Supplement: Supplementary file 1 — Appendix [file 41598_2017_9870_MOESM1_ESM.doc]

**The First Call Note Plays a Crucial Role in Frog Vocal Communication**

Journal name: Scientific Reports

**Xizi** **Yue^1, 2^, Yanzhu Fan^1^, Fei Xue^1^, Steven E. Brauth^3^, Yezhong Tang^1^, Guangzhan Fang^1,*^**

^1^ Department of Herpetology, Chengdu Institute of Biology, Chinese Academy of Sciences, No.9 Section 4, Renmin Nan Road, Chengdu, Sichuan, P. R. China

^2^ University of the Chinese Academy of Sciences, 19A Yuquan Road, Beijing, P. R. China

^3^ Department of Psychology, University of Maryland, College Park, MD 20742, USA

e-mail: [fanggz@cib.ac.cn](mailto:fanggz@cib.ac.cn)

**Supplementary Figure**

Supplementary figures for experimental designs and results of ERPs.


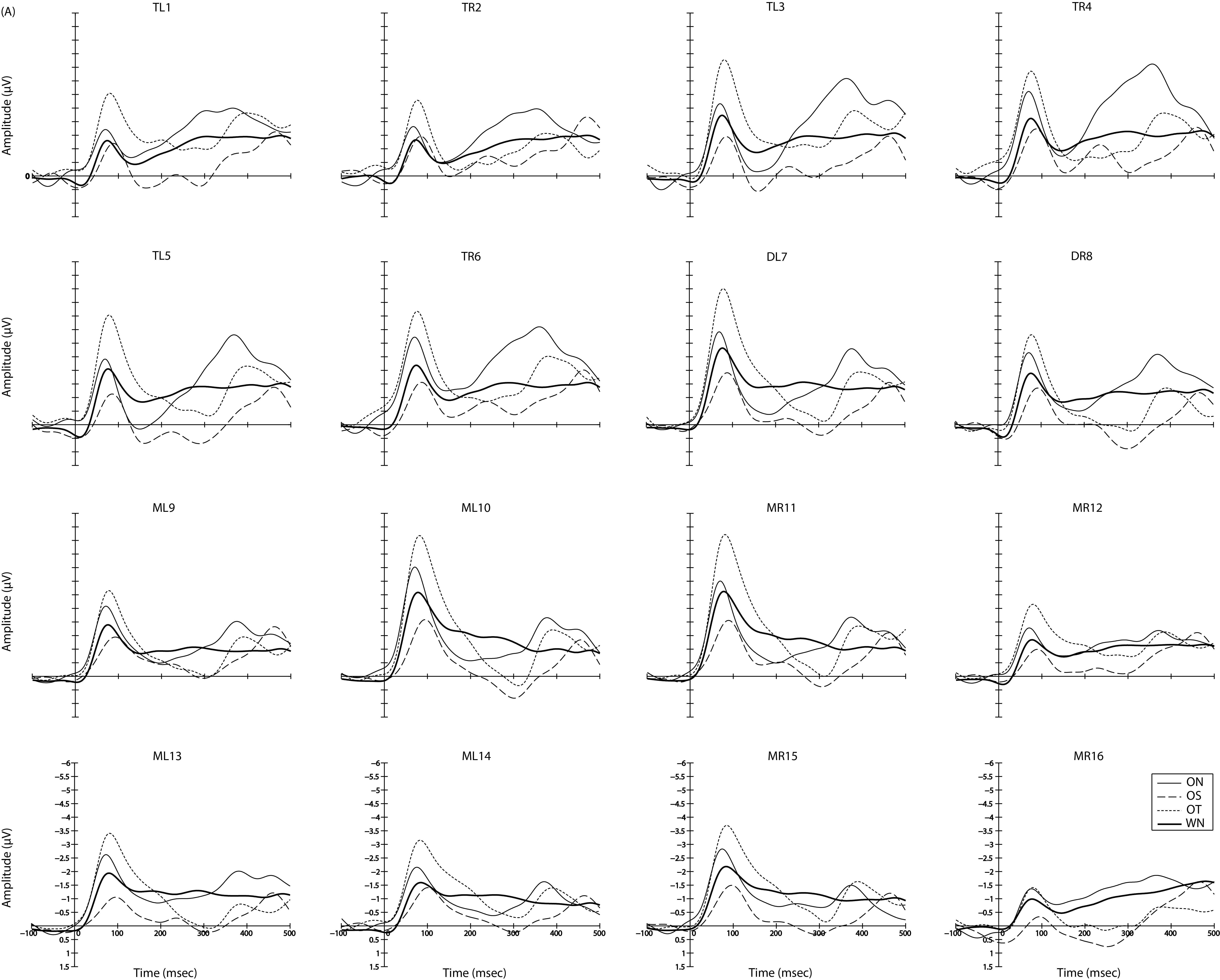


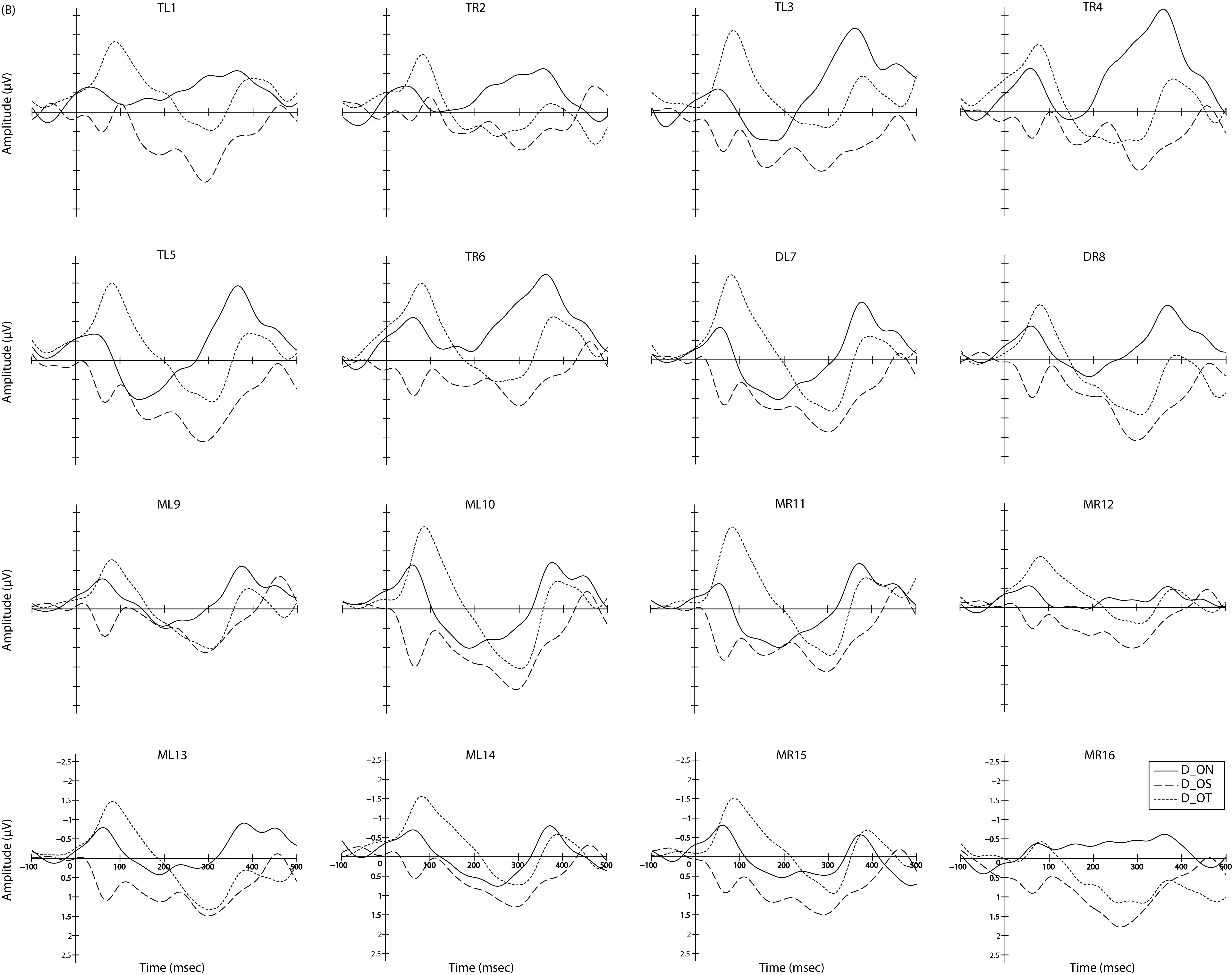


**Figure S1. The original waves for the four stimuli (A) and difference waves for the three deviant stimuli (B) with respect to each EEG channel.**

Abbreviation: ON, Original first note; OS, the reversed version of the first call note (only spectral characteristics remained unchanged, OS); OT, the envelope of the first call note filled by white noise (only the temporal characteristics remained unchanged, OT); WN, white noise; D_ON, D_OS and D_OT, the difference waves for the ON, OS and OT stimuli respectively.


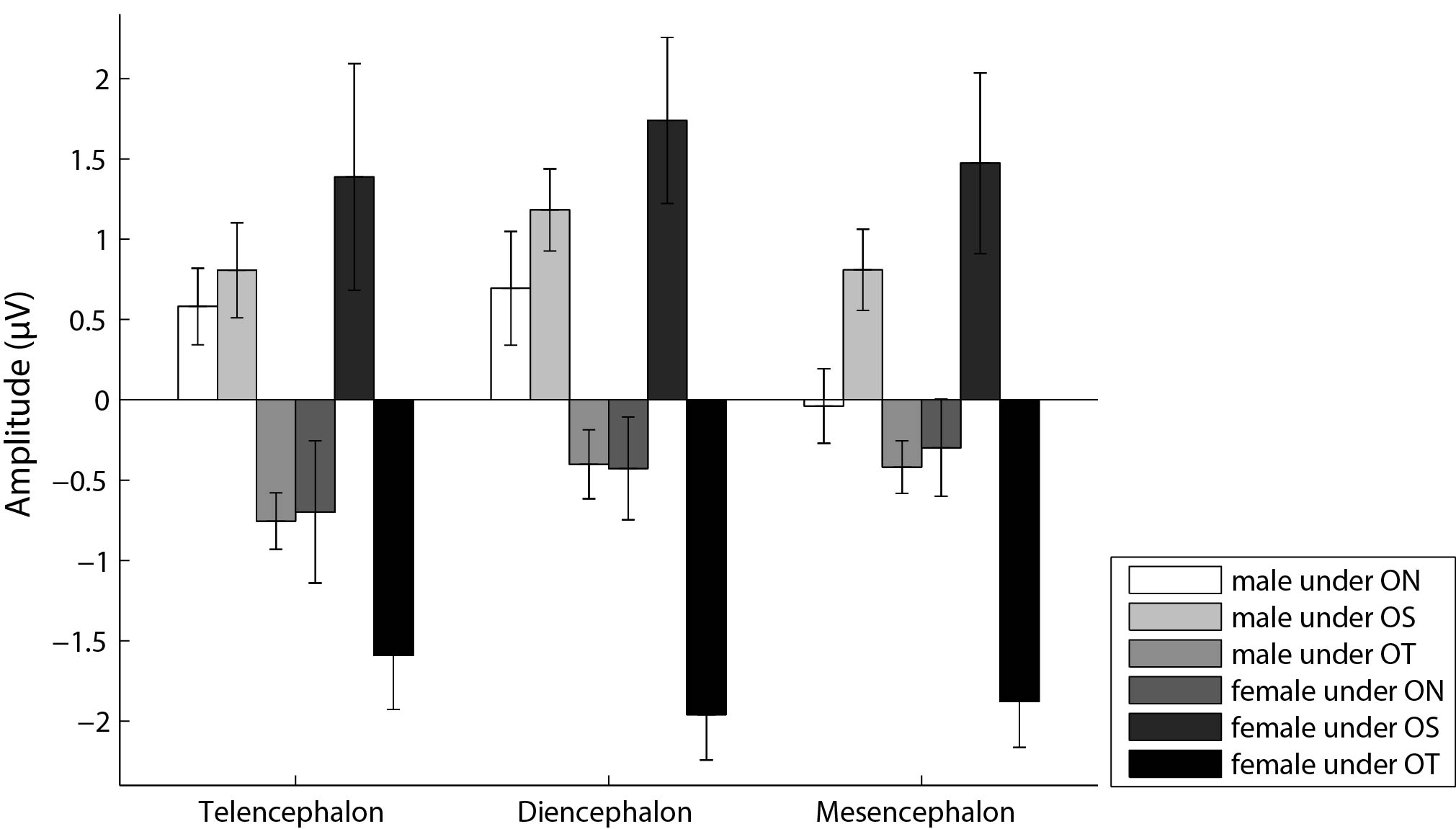


**Figure S2. The average amplitudes and standard errors of N1 difference waves for the telencephalon, diencephalon and mesencephalon respectively.**

Abbreviation: ON, Original first note; OS, the reversed version of the first call note (only spectral characteristics remained unchanged, OS); OT, the envelope of the first call note filled by white noise (only the temporal characteristics remained unchanged, OT).


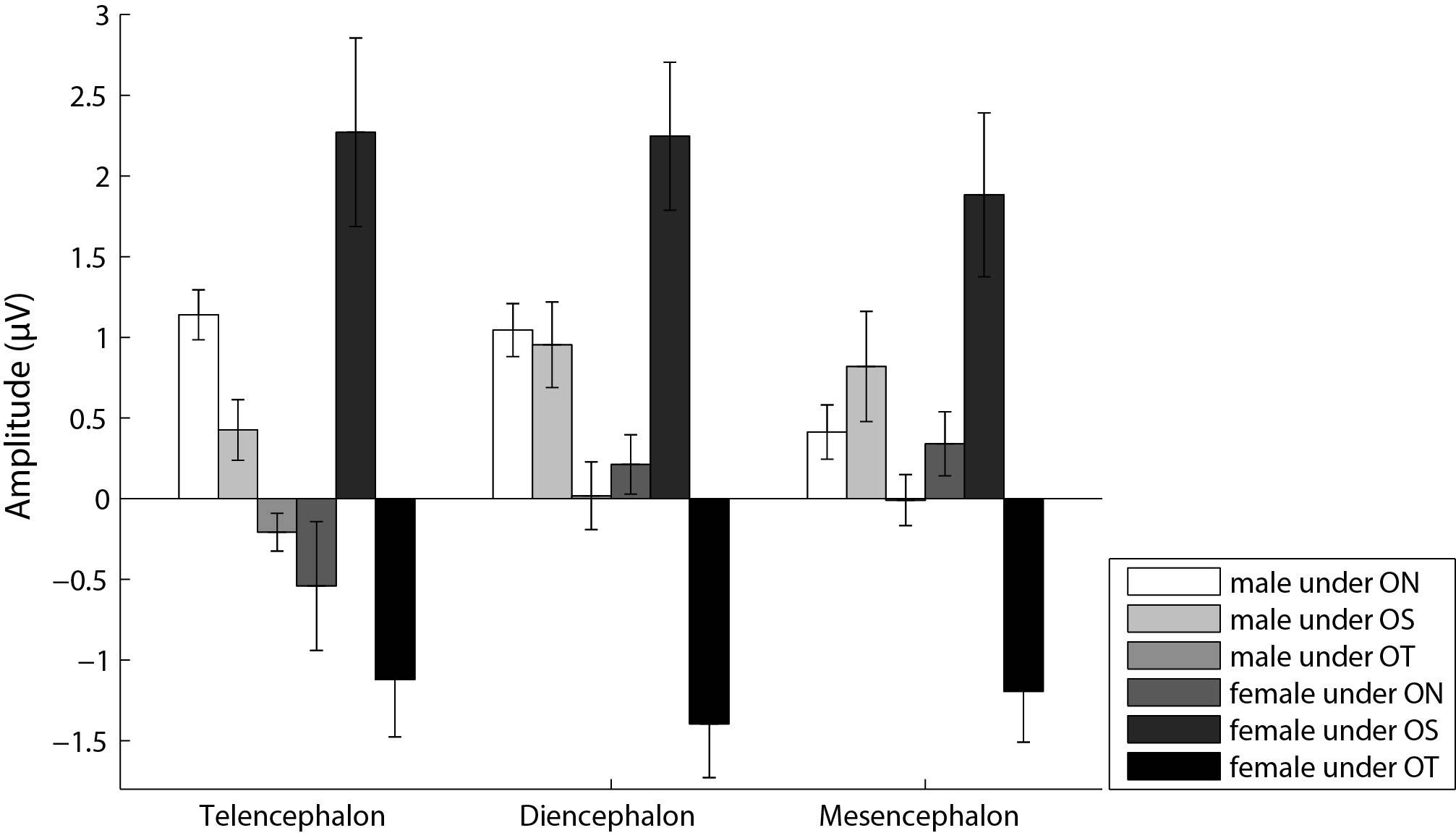


**Figure S3. The average amplitudes and standard errors of P2 difference waves for the telencephalon, diencephalon and mesencephalon respectively.**

Abbreviation: ON, Original first note; OS, the reversed version of the first call note (only spectral characteristics remained unchanged, OS); OT, the envelope of the first call note filled by white noise (only temporal characteristics remained unchanged, OT).


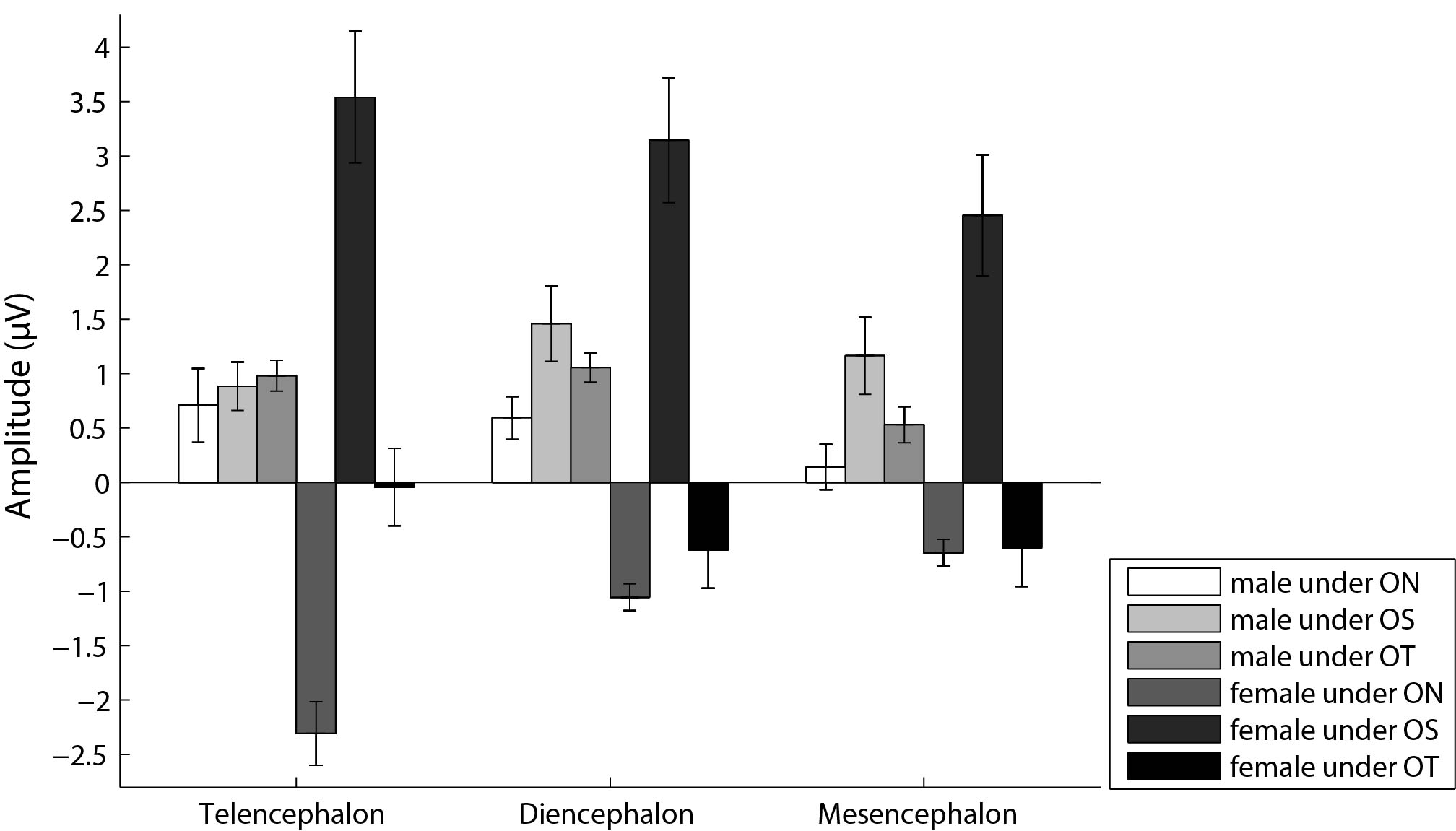


**Figure S4. The average amplitudes and standard errors of P3 difference waves for the telencephalon, diencephalon and mesencephalon respectively.**

Abbreviation: ON, Original first note; OS, the reversed version of the first call note (only spectral characteristics remained unchanged, OS); OT, the envelope of the first call note filled by white noise (only the temporal characteristics remained unchanged, OT).


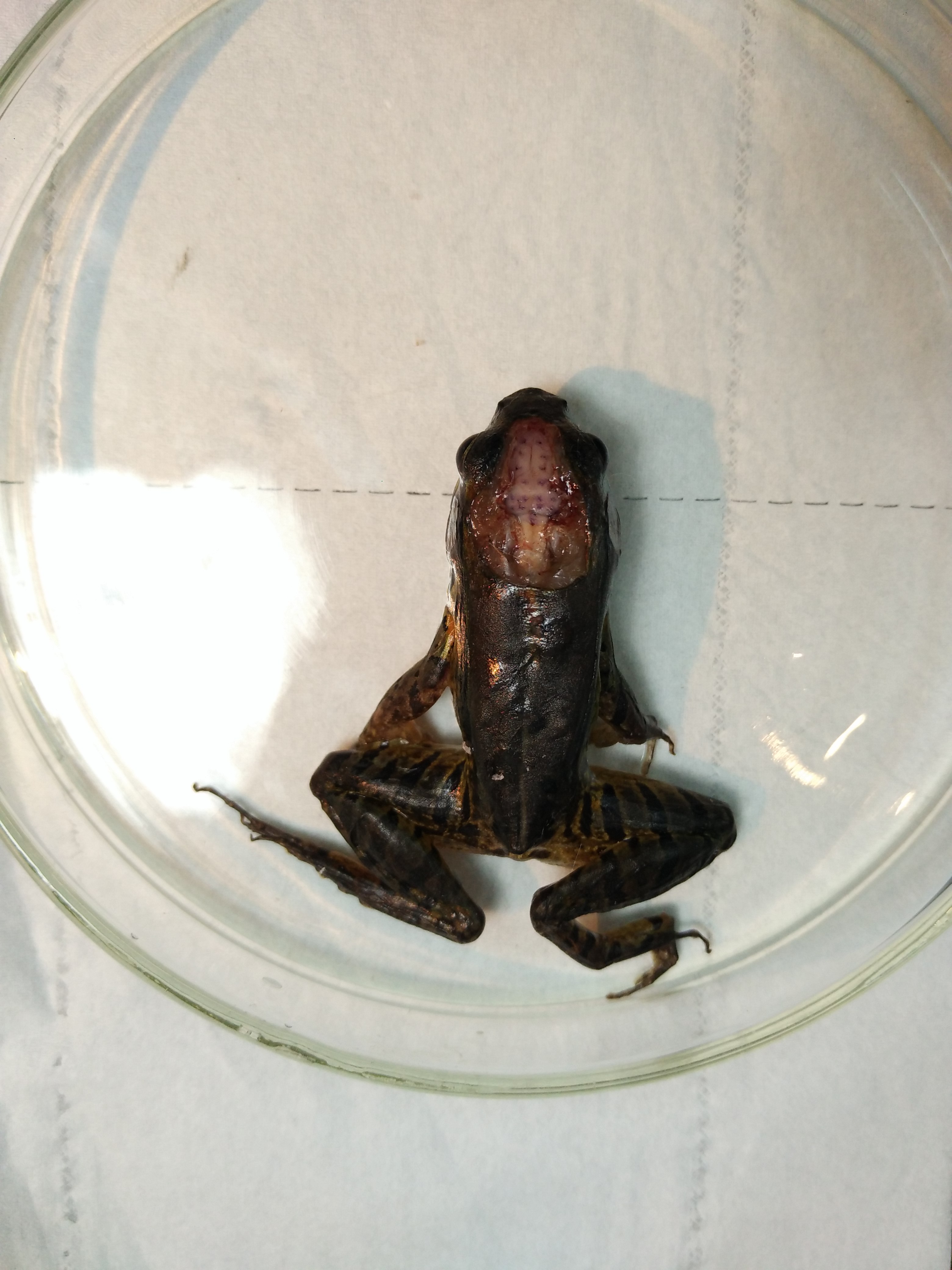


**Figure S5. An example showing the positions of 17 electrodes on the skull of a subject. There were 6, 2 and 8 electrodes above the telencephalon, diencephalon and mesencephalon respectively, while the reference was above the cerebellum.**

**
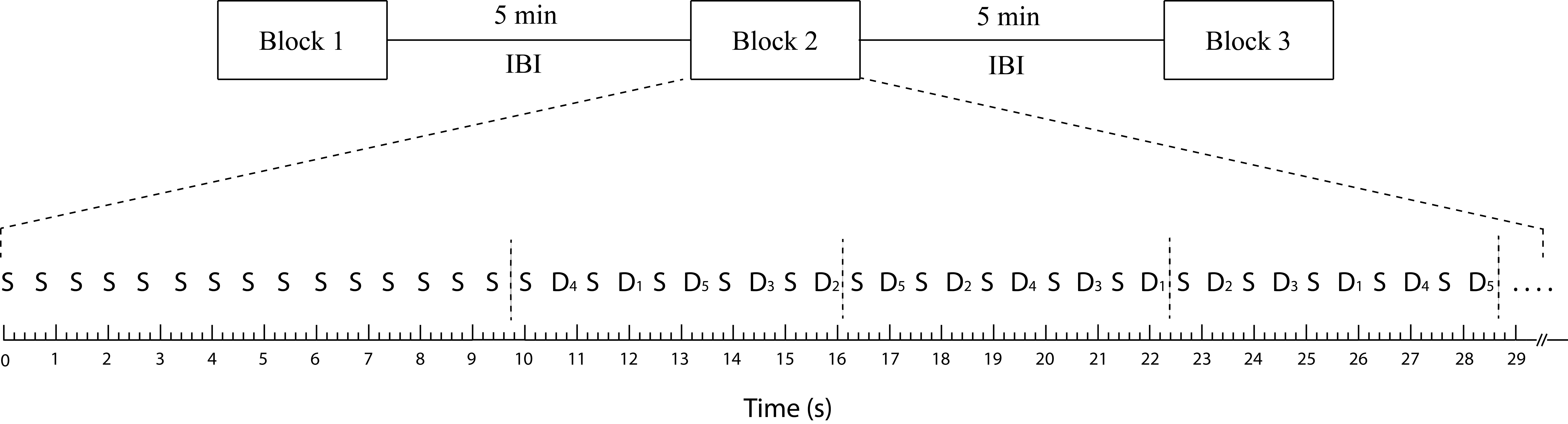
**

**Figure S6** A schematic diagram of the experimental design illustrating the temporal sequence of stimulus events and inter-stimulus intervals (ISI) within each block. The session was divided into three blocks with 5 min breaks between blocks. In each block, the first 15 acoustic stimuli were standards in order to strengthen the memory trace, and then one of the deviants was presented after each standard stimulus until all five deviant stimuli had been presented once, as shown between the two vertical dashed lines. IBI: inter-block interval; S: standard stimulus (white noise); D1-D5: the five deviant stimuli.


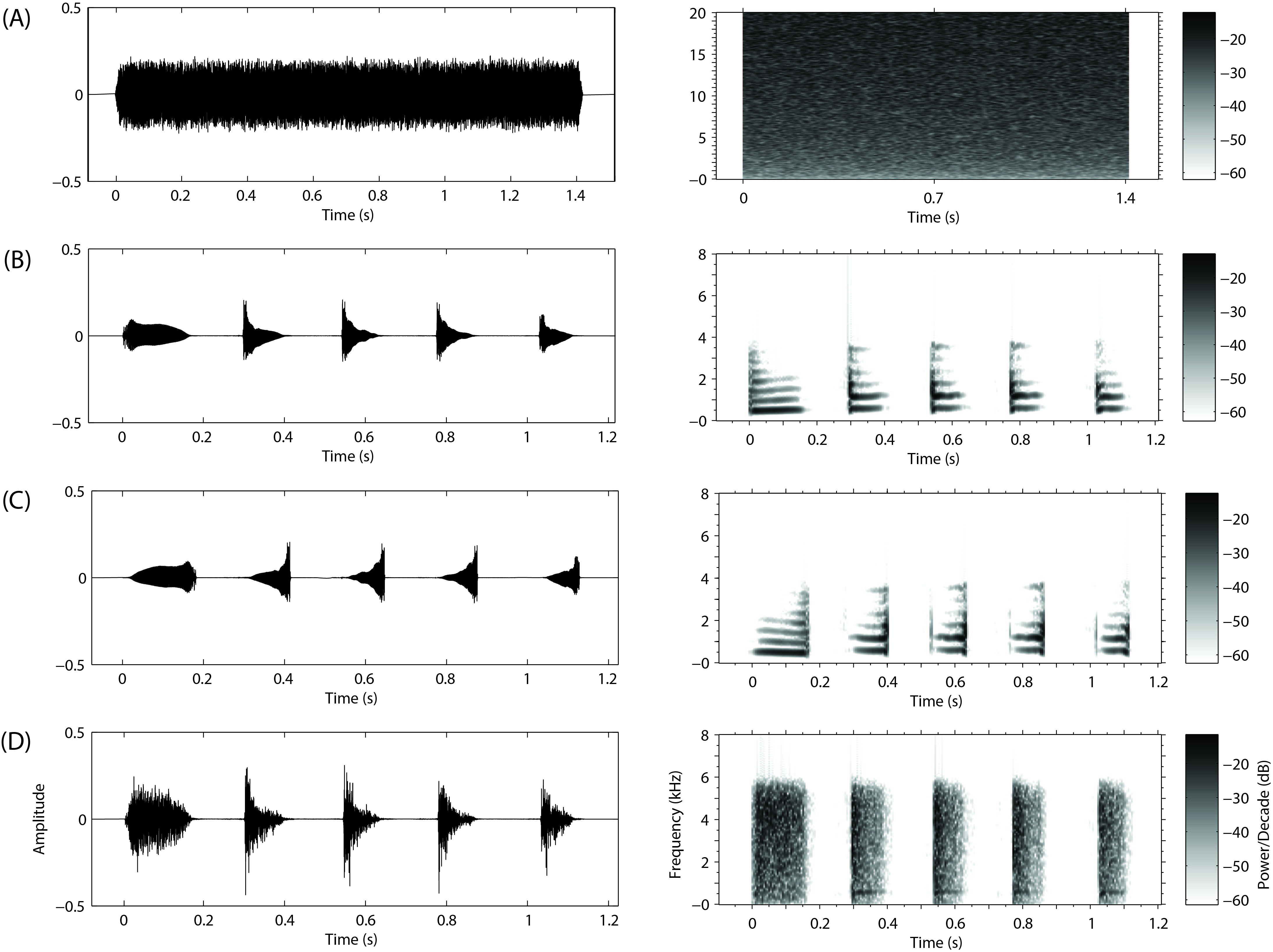


**Figure S7.** Waveforms and spectrograms of the four stimuli: (A) White noise; (B) the same advertisement call used in the MMN paradigm (Original notes, ON); (C) the reversed version of the advertisement call (only spectral characteristics remained unchanged, OS); (D) the envelope of the advertisement call filled by white noise (only the note temporal characteristics remained unchanged, OT).
